# Development of the Wits Face Database: an African database of high-resolution facial photographs and multimodal closed-circuit television (CCTV) recordings

**DOI:** 10.12688/f1000research.50887.1

**Published:** 2021-02-19

**Authors:** Nicholas Bacci, Joshua Davimes, Maryna Steyn, Nanette Briers

**Affiliations:** 1 Human Variation and Identification Research Unit (HVIRU), School of Anatomical Sciences, University of the Witwatersrand, Johannesburg, Gauteng, 2193, South Africa

**Keywords:** face database, CCTV, facial photographs, facial identification, facial comparison, morphological analysis, facial recognition

## Abstract

Forensic facial comparison is a commonly used, yet under-evaluated method employed in medicolegal contexts across the world. Testing the accuracy and reliability of facial comparisons requires large scale controlled and matching facial image databases. Databases that contain images of individuals on closed-circuit television (CCTV), with matching formal and informal photographs are needed for this type of research. Although many databases are available, the majority if not all are developed in order to improve facial recognition and face detection algorithms through machine learning, with very limited if any measure of standardisation. This paper aims to review the available databases and describe the development of a high resolution, standardised facial photograph and CCTV recording database of male Africans. The database is composed of a total of 6220 standardised and uncontrolled suboptimal facial photographs of 622 matching individuals in five different views, as well as corresponding CCTV footage of 334 individuals recorded under different realistic conditions. A detailed description of the composition and acquisition process of the database as well as its subdivisions and possible uses are provided. The challenges and limitations of developing this database are also highlighted, particularly with regard to obtaining CCTV video recordings and ethics for a database of faces. The application process to access the database is also briefly described.

## Introduction

Facial comparison is utilised by law enforcement to associate two sets of images, captured on video or photographically, to one another. Although different approaches exist, facial comparison by morphological analysis is currently considered the most reliable method ^1^. In this method, a target image, such as a snapshot from closed circuit television (CCTV) recordings obtained during criminal activity, and a standardised optimal image, such as a police mugshot, are compared to ascertain whether the two individuals are the same person. Facial comparison by morphological analysis has no directed standardised stepwise procedure as a validated methodology ^2^. Although facial comparison still does employ the analysis, comparison, evaluation and verification (ACE-V) tenets of forensic examinations ^1, 3^, its accuracy and reliability has not been tested extensively. As such, assessing its reliability should be considered a priority ^4^. The lack of validation can potentially be attributed to the logistic complexity required for rigorous scientific testing, of which a considerable limitation is the lack of standardised and actualistic databases to use ^5^. While several facial image databases exist (e.g. 5, 6), the incongruity of their composition is a major limiting factor.

The composition of many face image databases tends to be specific to the original intended use with a focus on various controlled conditions which makes them difficult to use for general purposes ^6^. For example, there is a variety of pre-landmarked databases available for use in the field of facial recognition (e.g. 7– 11) with many variations and controlled conditions. Some of the most commonly controlled-for conditions are orientation of the head/pose, illumination/lighting conditions, facial expressions, and age-related variations (e.g. 6, 7, 12). Despite the large number of databases available ( Table 1), there is a tendency towards either highly controlled data sets captured under very specific conditions with limited actualistic applications (e.g. 13, 14) or highly randomised images collected under inconsistent conditions (e.g. 7– 9, 15). In addition, most of these databases include a limited number of subjects with many replications under niche conditions and no standard baseline control images. Some of the datasets with a limited number of unique individuals include under 100 subjects (e.g. 13, 16, 17), with a handful including only 10 to 15 subjects (e.g. 14, 18– 20). Lastly, many of these databases are, by today’s standards, of subpar resolution with only two databases including images of resolutions greater than 640 x 480 pixels ^21, 22^. Between the highly specialised and varied conditions of capture, the lack of controlled images with matching realistic informal photographs of the same subjects, the low resolution, lack of methodological standardisation in image capture, and limited subject numbers, these facial image datasets provide very limited use in a forensic facial comparison context or more generalised facial studies.

**Table 1.** Overview of available face databases with available descriptives.

Database name and reference	No. of unique individuals	No. of images	Image resolution (pixels)	Database description and condition variations
AR Face Database ^30^	126 (70 Male, 56 Female)	4000	576 x 768	Various facial expressions, lighting, glasses, scarf
CVL Database ^31^	114	798	640 x 480	Various poses, varying facial expression
FERET Database ^32^	1199	14051	256 x 384	Various slight facial expressions, poses
Labelled Faces in the Wild (LFW) Database ^7^	5749	13233	250 x 250	Landmarked faces in various poses, expressions, lighting, ethnicity, age, clothing, hairstyles
Face Recognition Grand Challenge (FRGC) Database ^33^	688	Undefined	“High resolution”	Various facial expression, lighting
CAS-PEAL Face Database ^34^	1040 (595Male, 445 Female)	30900	360 x 480	Various poses, facial expressions, changes in lighting, glasses, caps
The MUCT Face Database ^29^	276	3755	640 x 480	Variations in pose, lighting, and annotated faces
The Yale Face Database ^18^	15	165	320 x 243	Facial expression variations, glasses
The Yale Face Database B ^19^	10	5760	640 x 480	Various poses and changes in lighting
CMU Pose, Illumination, and Expression PIE Database ^10^	68	41368	640 x 486	Various poses, facial expressions, changes in lighting, and glasses
Olivetti – Att – ORL ^35^	40	400	92 x 112	None
Japanese Female Facial Expression (JAFFE) Database ^20^	10	70	256 x 256	Various facial expressions
FIDENTIS 3D Face Database ^36^	2476	2476 complete 3D scans	12 megapixels	3D scans, landmarked, ear to ear facial pose equivalent
Caltech Occluded Face in the Wild (COFW) ^12^	Undisclosed	1852	Undisclosed	Various poses, expressions, lighting, occlusion focus, annotated
Ibug 300 Faces In-the-Wild (ibug 300W) Challenge database ^9^	600	>4000	Undisclosed	Various poses, expressions, lighting, annotated
Labeled Face Parts in the Wild (LFPW) Dataset ^11^	3000	3000	Undisclosed	Various poses, expressions, lighting
Quality labeled faces in the wild (QLFW) database ^8^	5749	277809	250 x 250	Various poses, expressions, lighting, ethnicity, age, clothing, hairstyles, distortions
Helen dataset ^37^	Undisclosed	2330	>500 width	Various poses, expressions, lighting, annotated
Facial expressions of emotion (KDEF) database ^17^	70 (35 Male, 35 Female)	4900	Undisclosed	Various facial expressions
NimStim facial expression database ^13^	43	672	Undisclosed	Various facial expressions
Annotated Facial Landmarks in the Wild (AFLW) database ^15^	25993 (11437 Male, 14556 Female)	21997	Undisclosed	Various poses, expressions, lighting, ethnicity, age, clothing, and hairstyles
Pointing Head Pose Image Database ^14^	15	2790	384 x 288	Various poses, glasses
BioID Database ^38^	23	1521	382 x 288	Various facial expressions, poses, lighting, accessories
University of Olulu Physics-Based Face Database ^39^	111	2112	428 x 569	Various poses (minor), lighting, glasses
Chicago Face Database ^22^	158 (73 Male, 85 Female)	Undisclosed	2444 x 1718	Various poses, facial expressions, lighting
SCface – Surveillance Cameras Face Database ^21^	130 (114 Male, 16 Female)	4160	Varied from 3072 x 2048 to 224 x 168	Various poses, normal and infrared
M2VTS multimodal face database ^16^	37	Minimum of 185	286 x 350	Videos and video to still images, various poses, some with and without eyeglasses, various time intervals (weeks)

Recently, databases consisting of video recordings of faces have become more common (e.g. 21, 23– 26) due to the increase in CCTV surveillance cameras. Generally, these databases have either been collated *via* recording of known participants in controlled environments (e.g. 21, 23, 26), or were based on pre-recorded videos obtained from various media on the internet or movies ^24, 25^. To the best of the authors’ knowledge, only a single other database ( SCface – Surveillance Cameras Face Database) published includes both facial photographs of high resolution and corresponding still images extracted from CCTV recordings of varying resolutions ^21^. These databases are primarily used in testing and developing head/face detection and tracking or automated face annotation systems under a variety of video recording conditions.

The majority of facial image databases are primarily or exclusively inclusive of males of either European or East Asian descent (e.g. 17, 27, 28). Only a few databases contain individuals from other ancestry groups, particularly African individuals (e.g. 6, 7, 13), and they have a limited number of individuals. This is evident when considering there is only a single other South African face database ^29^. Although a great initiative for a large, landmarked face database specifically developed to increase the variety of lighting, ethnicity and age available, it consists primarily of low-resolution webcam-based images with often very distorted lighting conditions. In addition, a total of 276 subjects were used for this database with no control images or demographic specifications provided.

The majority of existing facial databases have been developed for the purposes of facial recognition and machine learning training and do not contain target and control images of the same individual. These databases tend to be sourced from public, internet images containing faces in a variety of inconsistent conditions (e.g. 9, 11, 12). To the best of the authors’ knowledge, no databases exist that are intended specifically for use with facial comparison by morphological analysis. As such, we aimed at creating a system for developing a consistent face database with corresponding individuals across various photographic and video recording conditions, resulting in the Wits Face Database (WFD). This database is intended to be a functional, actualistic African database of facial images that can be utilised for facial comparison analyses and research in craniofacial identification. This database is intended to be a free resource strictly for non-commercial scientific research, provided access has been cleared by an ethics committee overseeing its use.

## Materials and methods

The database was established by collecting photographs of willing participants on the University of the Witwatersrand campus, Johannesburg, South Africa, including corresponding CCTV recordings. The database is comprised of CCTV recordings and photographs gathered on university premises, between July 2018 and October 2019, *via* the pre-existing CCTV systems used by campus security. Facial photographs were standardised and in five different views. The CCTV recordings were captured in a variety of conditions, such as from different quality cameras, in different formats and heights of recording, and with disguises (sunglasses and caps).

### Ethics and participant recruitment

Ethical approval was obtained from the Human Research Ethics Committee (Medical) of the University of the Witwatersrand (clearance certificate No.: M171026). Permits from the campus head of security and deputy registrar were obtained prior to data capture, in their capacity as site managers. Facial photographs were captured by an experienced photographer following recruitment of participants. CCTV recordings of matched participants were collected through the university’s security systems. The original target of participants intended for the database were 600. However, due to data loss as a result of power failures and data transfer corruption a greater number of participants had to be recruited for redundancy purposes. Potential participants were identified and recruited near the data collection sites based on facial anthropological features resembling males of South African descent and being older than 18 years of age. Approached participants were then informed of the greater project orally and with an information sheet ^40^ and asked for voluntary participation in the study and database. Once agreed, they signed an informed consent form ^40^ prior to being photographed or recorded. If participants requested to be removed from the database, all their photographs and recordings were erased and their recruitment information shredded. None of the personal details and images given by the participants are to be freely distributed. As agreed with the ethics committee, and according to the consent forms signed by the participants, no identifiable images of any individuals are to be published without following up with participants for additional consent. As a result, for publication purposes to indicate examples of the face database, one of the author’s images have been utilised.

### Image acquisition

Facial photographs and recordings were collected at three designated access-controlled locations with a large influx of potential participants on the Braamfontein Campus of the University of the Witwatersrand, Johannesburg, South Africa. The following three collection sites were set-up and utilised:

- Site A: outdoors CCTV camera (installation height: 3100 mm) with a view of a student card terminal near the food concourse and Student Union Building, the Matrix.
- Site B: outdoors CCTV camera positioned at eye-level height (1700 mm) in one of the more frequented pedestrian entrances in proximity to the Oppenheimer Life Sciences building.
- Site C: indoors analogue CCTV camera (installation height: 2500 mm) in the administration building concourse in view of the cashier’s offices, Solomon Mahlangu House.

**Figure 1.**
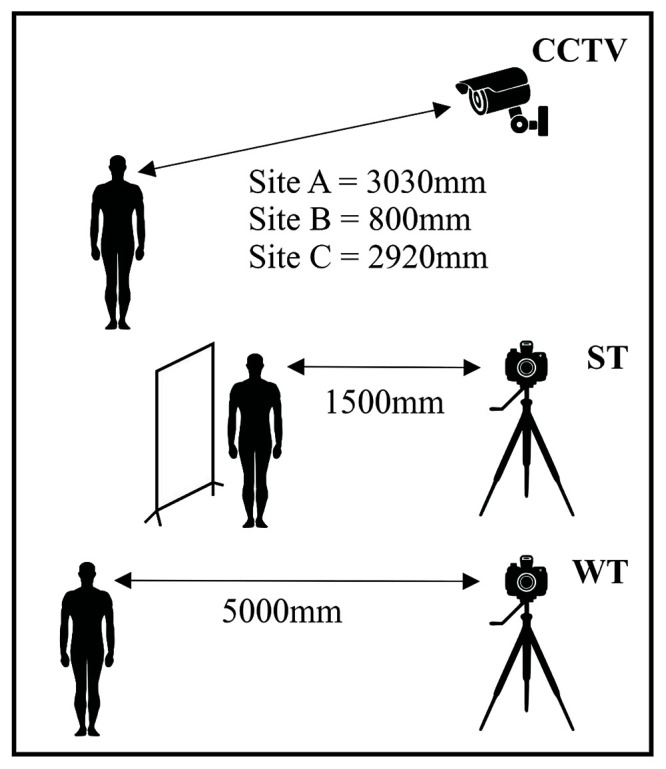
Schematic diagram of camera set-up for closed-circuit television (CCTV) and facial photograph capture. ST = standardized photograph; WT = wildtype photograph.

**Figure 2.**
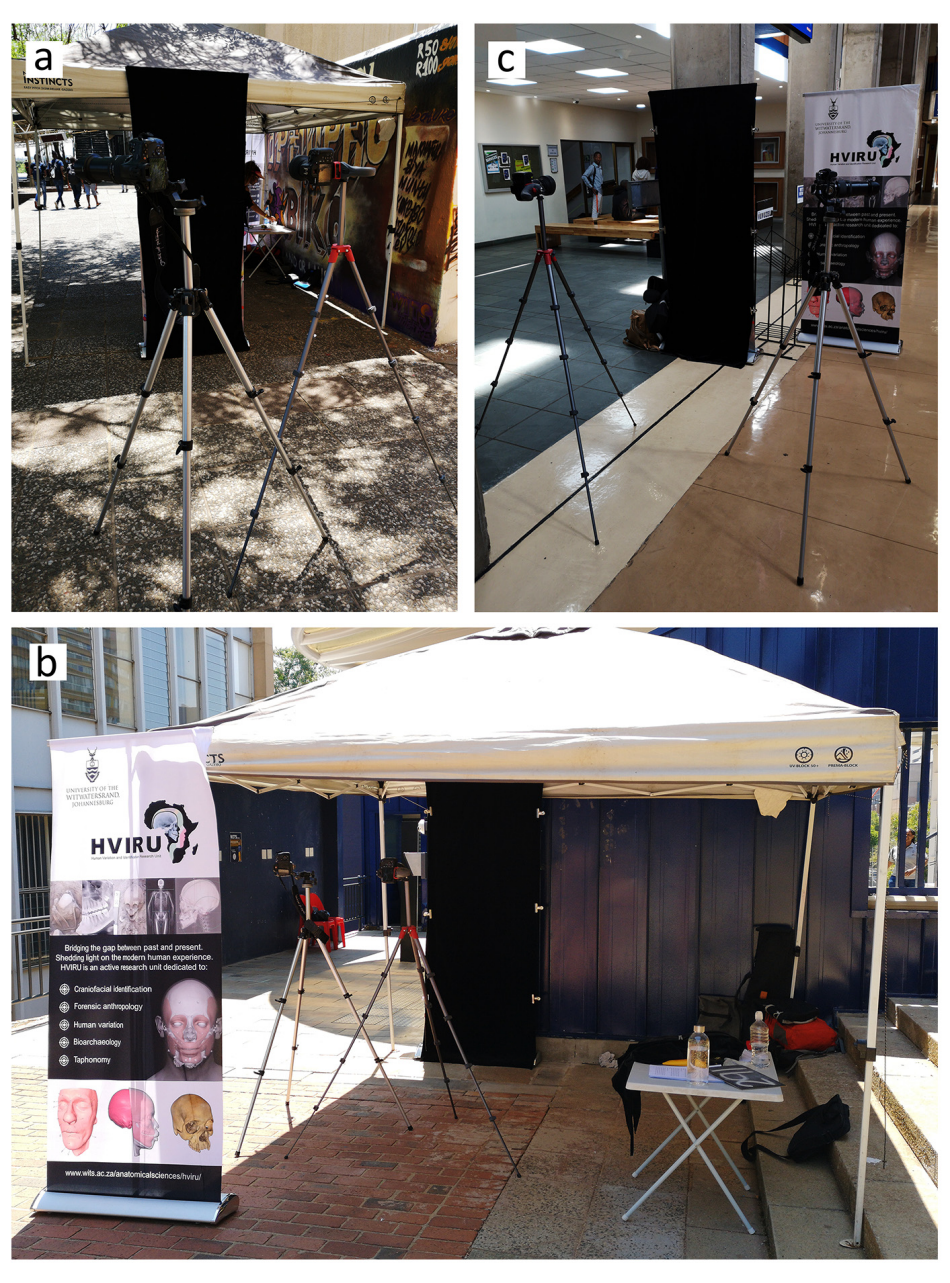
Actual photographic camera set-up in the process of database development. Arrangement of cameras and backdrop for standardised and wildtype photographs at site A ( **a**), site B ( **b**) and site C ( **c**).

In the vicinity of the installed CCTV cameras at each site, a photography station was set up in a standard manner as demonstrated in Figure 1 and Figure 2. Within this set-up, facial photographs were captured in five different views using two different cameras with slightly different parameters and conditions. All cameras were arranged in a marked fixed location near the CCTV field of view on tripods at a fixed height of 1600 mm. This height was maintained to attempt centring the field of view of the photographs on the face, as the mean height for black South African males ^41^ is 1710 mm. Specifically, the eye level of each participant was composed on the top horizontal rule of thirds line for all photographs taken. All photographs were taken in portrait orientation.

An example of the arrangements for database image capture and recording on pre-existing CCTV systems is shown in Figure 2. Distances were controlled for participants to be photographed and recorded at each site. Standardised (ST) photographs, with a solid black backdrop and participant clothing covered, were captured at a distance of 1500 mm. Wildtype (WT) photographs were captured at a 5000 mm distance. These photographs included a mixed background that was intentionally meant to simulate real-life photographic conditions. WT photographs were taken in a simulated scenario of suboptimal facial images with a comparable quality camera and facial poses. The background was purposefully not controlled for with a mixed environment visible and varied based on the location site of data collection. This background was intentionally out of focus to simulate suboptimal photographic conditions. Despite a minor level of variation, consistency was maintained across all photographs with regard to distance to subject, aperture, and composition.

**Figure 3.**
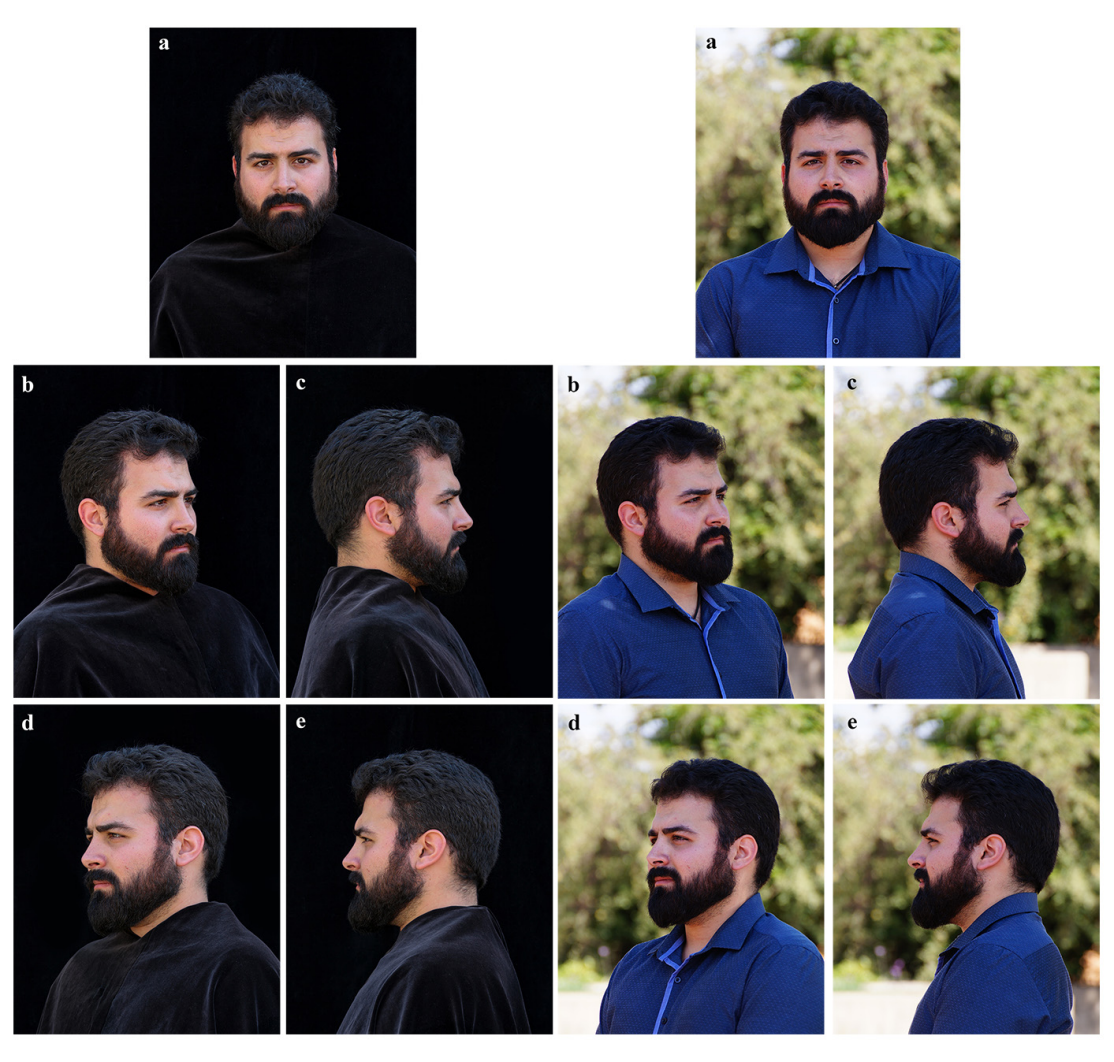
Example of the five views of standardised (left) and wildtype (right) facial photographs captured. The five views of facial photographs are demonstrated in this image, showing anterior ( **a**), right 45-degree ( **b**), right lateral ( **c**), left 45-degree ( **d**) and left lateral ( **e**) views.

The first set of photographs were captured under standardised conditions using a Canon 1300D 18MP DSLR camera (18–55 mm DC Canon lens) with the following settings: image sensor sensitivity (ISO) of 800, aperture F/9, shutter speed between 1/125 and 1/40, focal length of 55 mm and daylight white balance. For these standard photographs, the objective to face distance was fixed at 1500 mm. A set of standardised photographs were taken, with a black backdrop and with the participants’ clothing covered by a black velvet cloth – similar to the backdrop – in order to prevent matching participants based on clothing appearance. These standard photographs were captured in the following five views ( Figure 3):

a. Anterior frontal view
b. Right 45-degree view
c. Right lateral view
d. Left 45-degree view
e. Left lateral view

The second set of WT photographs captured for each participant, corresponded to the same views as described above, using a Sony SLT A57 (18 – 250 mm Sony Zoom Lens) with the following settings: image sensor sensitivity (ISO) of 200, aperture F/9, shutter speed between 1/125 and 1/40, focal length of 250 mm and daylight white balance. Indoor photographs at site C were captured at a range of ISOs between 400 and 1600 depending on the varying light conditions.

All photographs were captured as both native .jpeg and RAW format. Images were then downloaded from the SD cards of each camera, stored, and sorted on a desktop computer. All images were then batch cropped at a 4x5 (8x10) aspect ratio, using Adobe Photoshop CS6, to only include the participants as centred in the frame of the photograph. The resulting image resolution for the ST photographs was 3456 x 4320 pixels at 300 dpi and sRGB colour representation. The WT photographs’ resolution was 3264 x 4080 at 350 dpi and with sRGB colour representation. Following batch cropping, the standardised images were imported into Adobe Lightroom (v. 5.3) for basic editing. The only adjustments made included exposure level, highlights, and shadow correction. Additionally, removal/spot healing of any exposed clothing or background features were done if the cloth or backdrop did not fully obscure the participant and surroundings. The wildtype images were left unaltered post cropping. The above discussed image processing (batch cropping) and adjustments (exposure level, highlights, shadows correction and spot healing) can alternatively be performed using open source software such as GNU Image Manipulation Program ( GIMP), Photivo, and darktable.

CCTV recordings from internet protocol (IP) cameras were transferred live to a HikVision (model: DS-9664NI-I16) server for storage. Videos from the analogue camera were stored on a DS-ENC-V120B20121026-7054D2ABA6FB digital video recorder (DVR) device. All recordings were then extracted in .mp4 format from the University of the Witwatersrand’s CCTV systems through the university’s protection services software ( iVMS 4200 v. 2.71.9). Only footage recorded during data collection times was extracted from the CCTV systems. During recording, participants were asked to stand at a marked location and rotate through the same five views that the photographs were captured in for approximately 5 seconds. Following this, the participant acted out the process of utilising a student card terminal in view of the camera (sites A and C) or attempting to exit the campus (site B).

**Figure 4.**
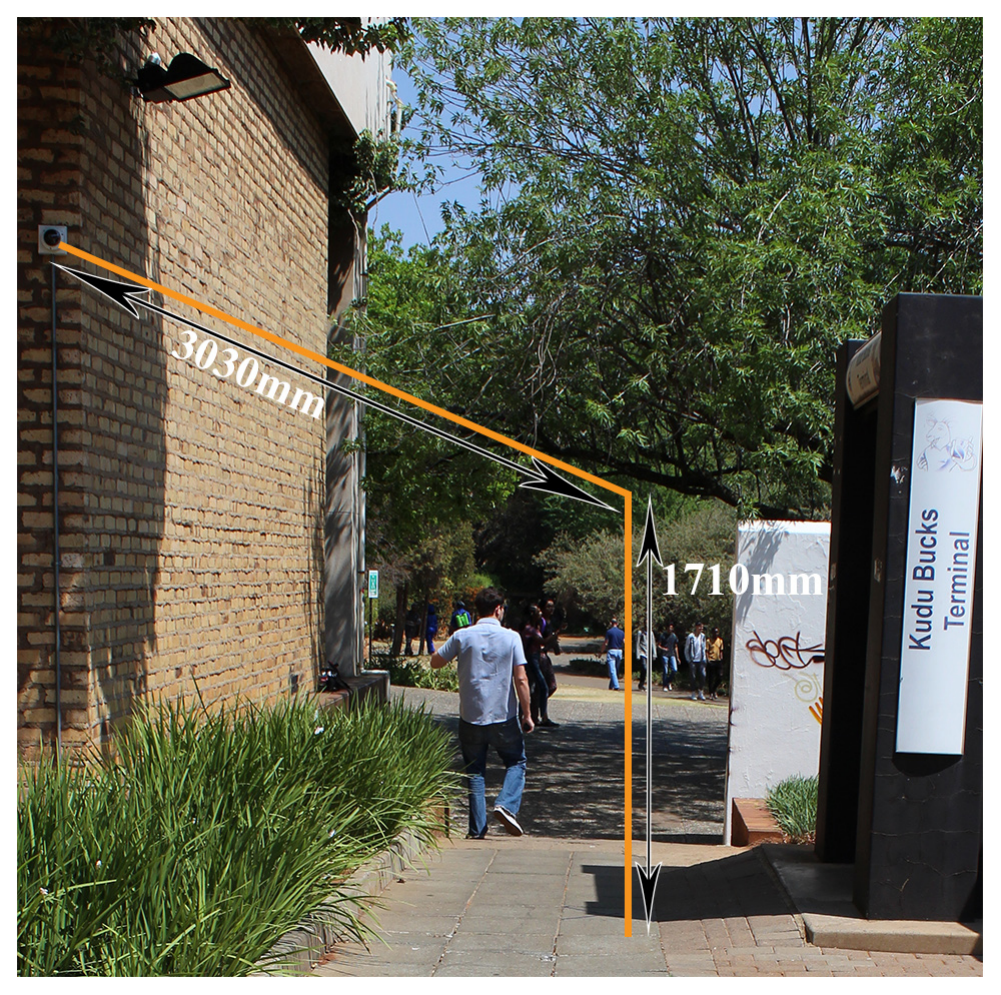
Demonstration of estimated objective to face distance at site A. Vertical distance indicates the mean height of a South African male (1710 mm) and the oblique distance indicates the calculated approxiamte camera to face distance at Site A (3030 mm).

The outdoor CCTV camera at site A was an IP camera (HikVision, model: DS-2CD2142FWD-I, 4-megapixel, 4 mm fixed lens, aperture F/2). This camera was installed at a height of 3100 mm and a floor distance of 2690 mm from the marked location where participants stood in the process of video capturing. The resulting distance between the participants’ face and the IP camera objective was 3030 mm ( Figure 4). This distance was calculated based on the mean height of the average black South African male of 1710 mm ^41^. The angle of incidence from the camera objective to the face was approximately 27°.

The outdoor “eye-level” IP CCTV camera at site B had the same model and specifications of the camera at site A and was installed at a height of 1700 mm from the ground, marked location to floor distance of 800 mm. The resulting distance from target face to camera was 800 mm and the angle of incidence was virtually zero.

The indoor analogue CCTV camera at site C (Securi-Prod 1/3” Sony Effio E 700TVL indoor dome, model: CC217, 2.8 – 12 mm vari-focal lens) was installed at a height of 2500 mm and at a floor distance from the marked area of 2810 mm. The resulting distance between camera and face was 2920 mm and the angle of incidence was approximately 22°.

**Figure 5.**
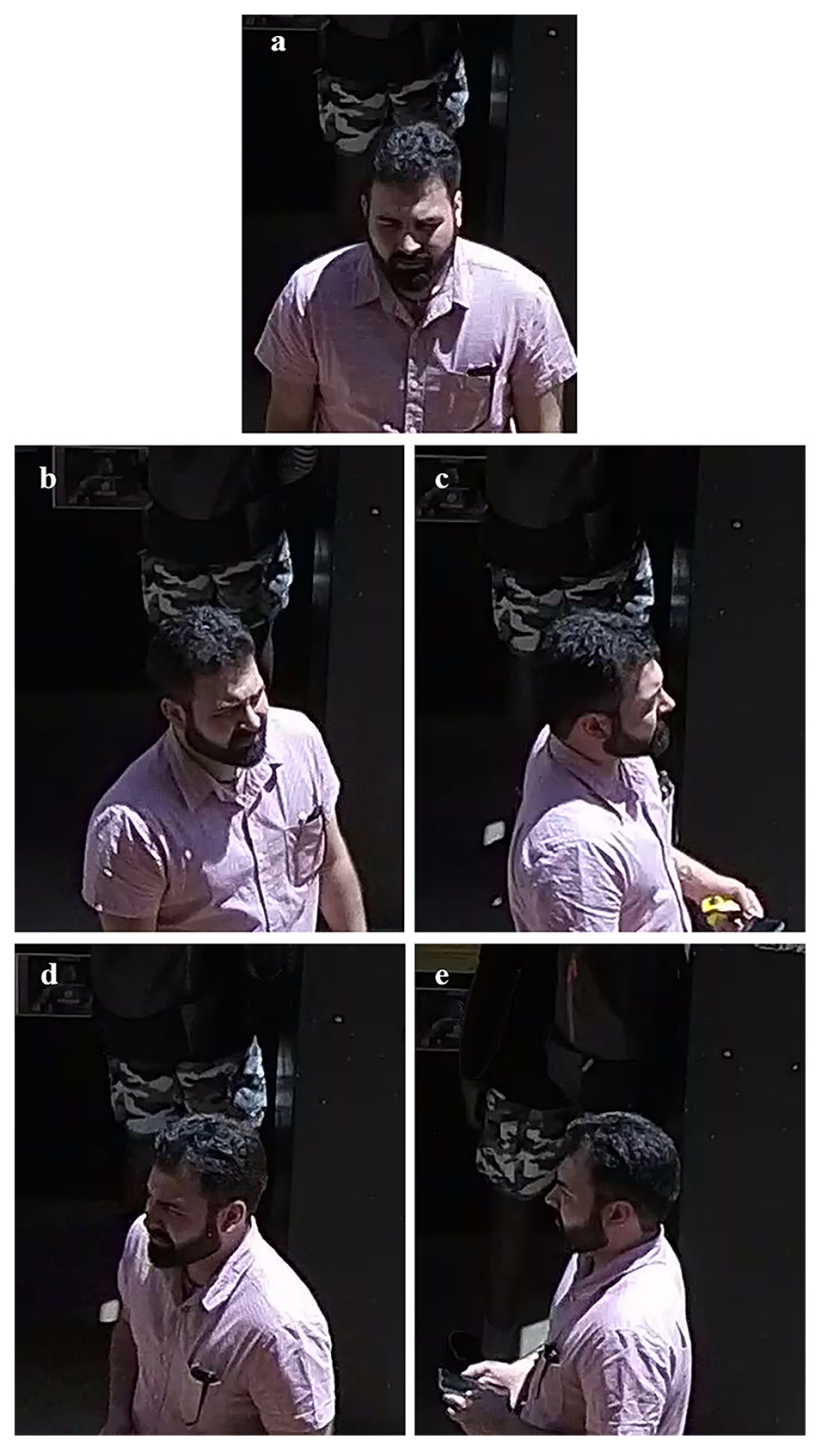
Example of the five views of standard closed-circuit television (CCTV) stills captured at site A. The five views of facial photographs are demonstrated in this image, showing anterior ( **a**), right 45-degree ( **b**), right lateral ( **c**), left 45-degree ( **d**) and left lateral ( **e**) views.

**Figure 6.**
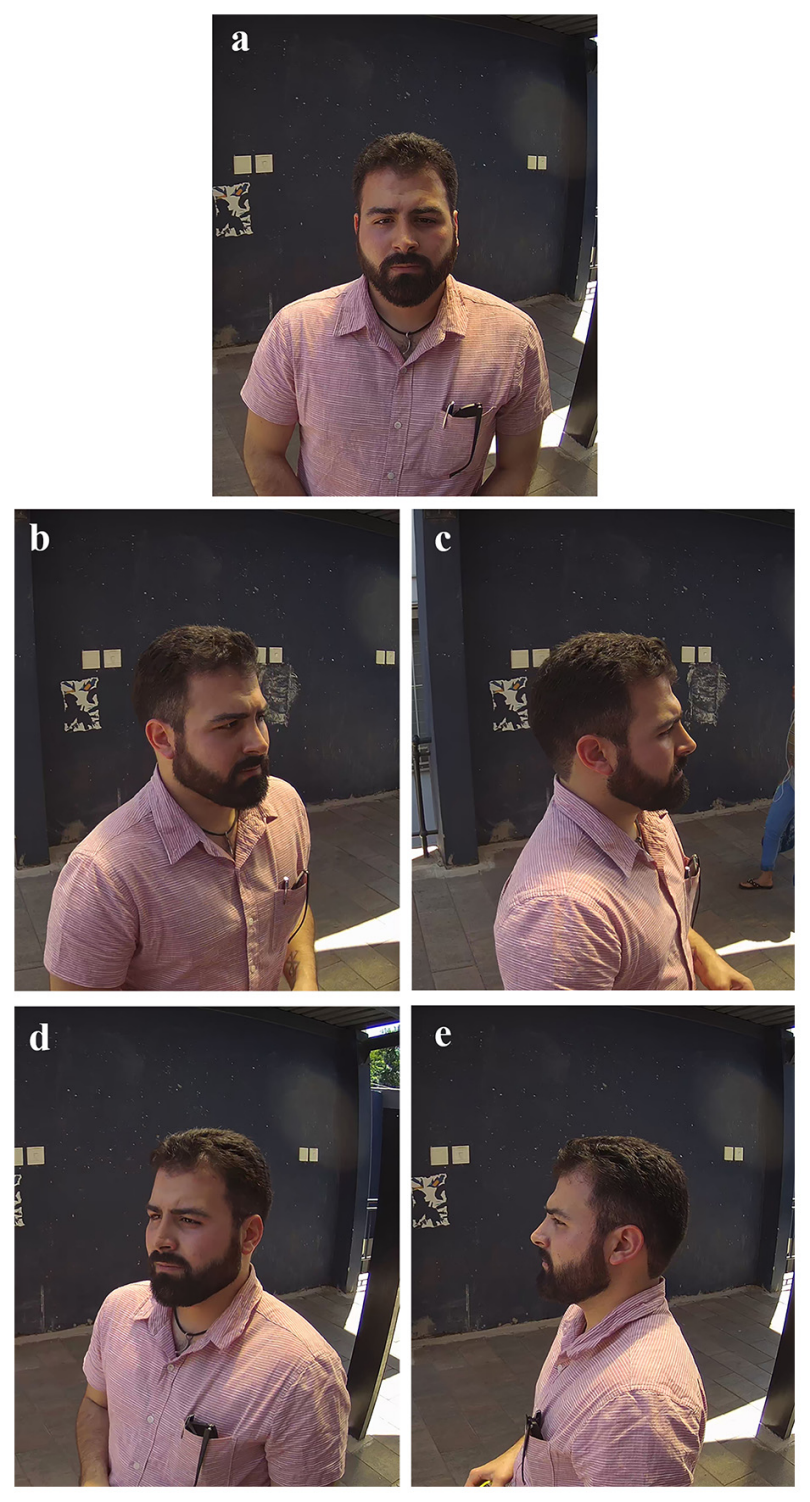
Example of the five views of eye level closed-circuit television (CCTV) stills captured at site B. The five views of facial photographs are demonstrated in this image, showcasing anterior ( **a**), right 45-degree ( **b**), right lateral ( **c**), left 45-degree ( **d**) and left lateral ( **e**) views.

**Figure 7.**
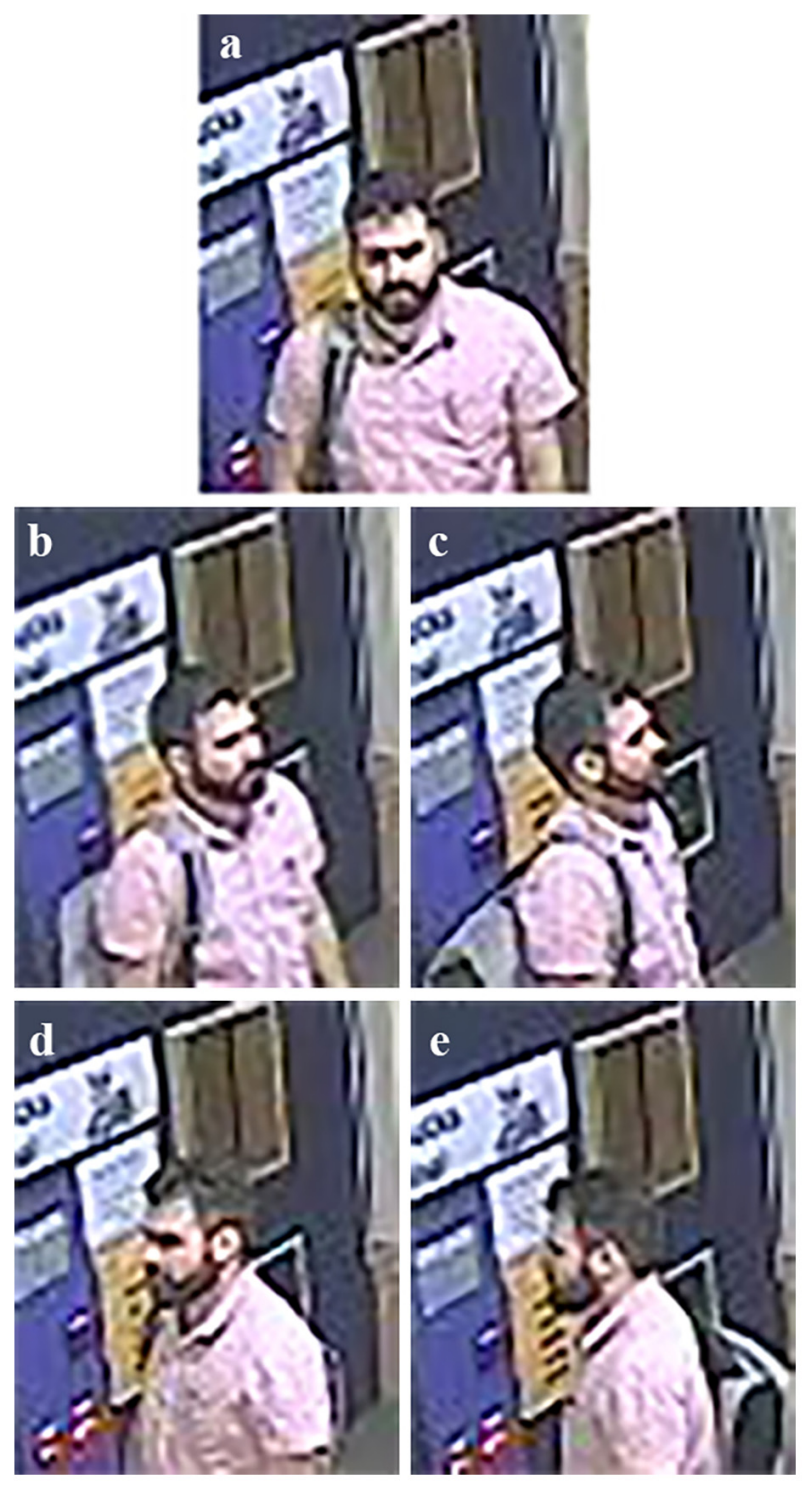
Example of the five views of analogue closed-circuit television (CCTV) stills captured at site C. The five views of facial photographs are demonstrated in this image, showing anterior ( **a**), right 45-degree ( **b**), right lateral ( **c**), left 45-degree ( **d**) and left lateral ( **e**) views.

**Figure 8.**
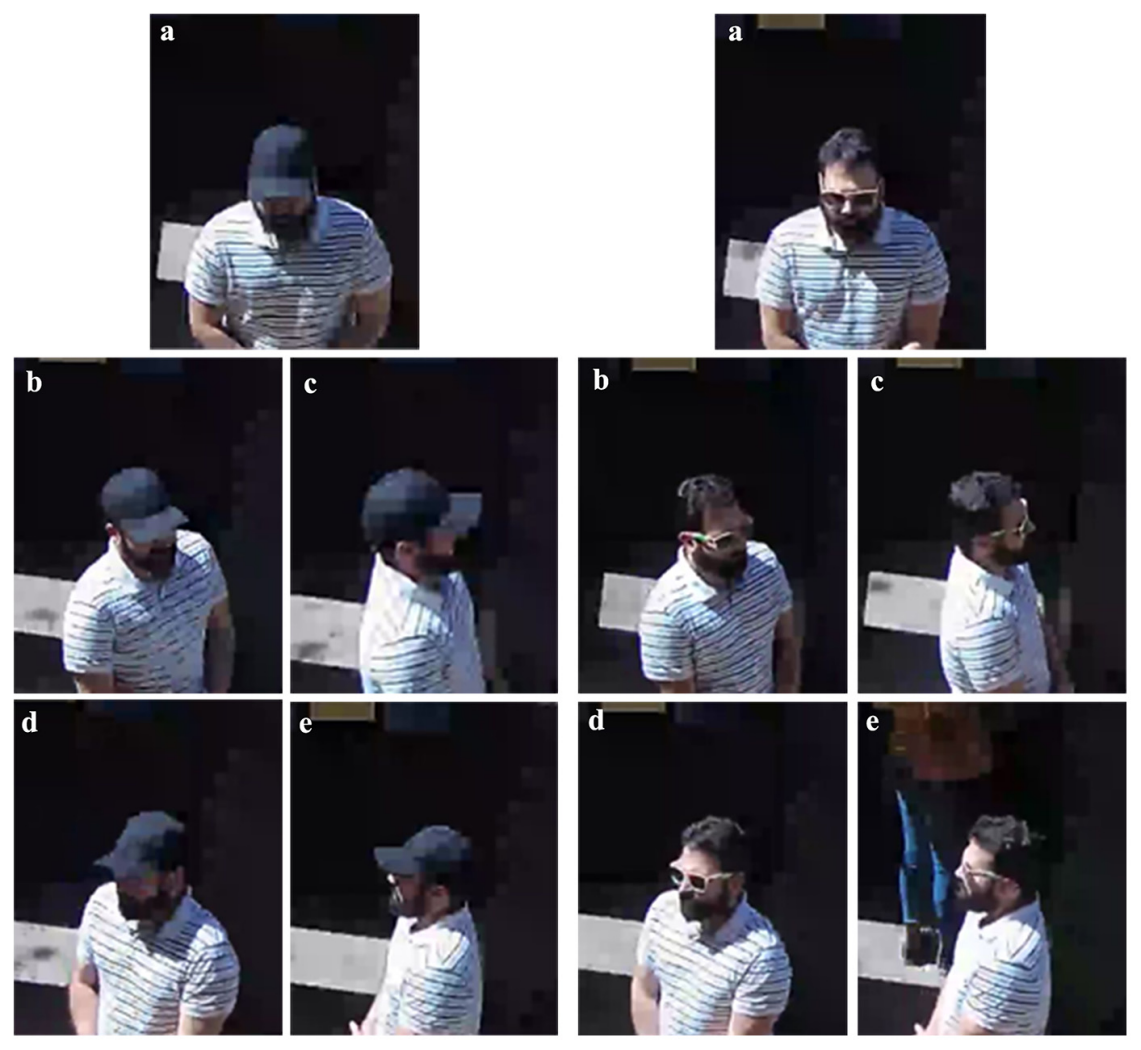
Example of the five views of closed-circuit television (CCTV) recordings with two obstruction types - brimmed cap (left) and sunglasses (right). The five views of facial photographs are demonstrated in this image, showing anterior ( **a**), right 45-degree ( **b**), right lateral ( **c**), left 45-degree ( **d**) and left lateral ( **e**) views.

A total of approximately 30 seconds of footage was recorded for each participant and out of the 30 seconds five still images were captured from the footage at each of the five views previously described ( Figure 5, Figure 6, Figure 7 and Figure 8). The photographs and recordings of each individual were coded with a participant number to maintain anonymity of the participants and a separate record of the identity of the participants was retained. Complete anonymity of the participants included in the facial image database was maintained. Images and videos included in the database were stored on three separate password protected desktop computers and encrypted on a constantly monitored external hard disk drive. In addition, the dataset was also transferred to the University of the Witwatersrand’s Library repository under restricted access to data management services. This repository retains the database in triplicate for cataloguing and in order to allocate searchable metadata to it to facilitate use.

## Database composition

The database was composed strictly of male South Africans of African descent. Participants were over the age of 18, for consent purposes, and in the young adult age-ranges, between 18 and 35 years of age. The participants were subdivided into a series of cohorts depending on the type of matched analysis possible, as outlined in Table 2. All participants were photographed in both the standardised and wildtype setting at the various sites. The first two groups were only photographed under natural outdoor lighting at site A (n=120) as well as with artificial indoor fluorescent lighting at site C (n=99). For each participant 10 photographs were captured, totalling 1200 photographs under outdoor conditions with natural lighting conditions and 990 photographs under indoor fluorescent lighting conditions. A second group, in addition to being photographed as above, was recorded on an outdoors setting CCTV camera at site A (n=98, n=86 with corresponding footage). A third group was recorded at site B with the eye-level camera (n=108, n=76 with corresponding footage). A fourth cohort was recorded at site C with the analogue CCTV camera and lastly a final group of participants were recorded with obstructive accessories, namely caps (n=45, n=34 with corresponding footage) or sunglasses (n=41, n=31 with corresponding footage), using the same IP camera at site A. Due to data corruption or data loss in the CCTV recording process, not all photographed individuals have corresponding footage associated to them. Overall, the database is inclusive of over 6200 facial photographs and 334 corresponding video recordings from various types of CCTV cameras within an African sample ( Table 2).

**Table 2.** Detailed categorisation of Wits Face Database (WFD) composition by cohorts of potential analyses.

Cohort of matching individuals	No. of participants	No. of photographs	Participants with corresponding CCTV footage
*Photo-Photo Outdoor (Site A)*	120	1200	0
*Photo-Photo Indoor (Site C)*	99	990	0
***Photo-Photo Totals***	**219**	**2190**	**0**
*Photo-CCTV Outdoor (Site A)*	98	980	89
*Photo-CCTV Outdoor Eye Level (Site B)*	108	1080	76
*Photo-CCTV Indoor Analogue (Site C)*	111	1110	107
*Photo-CCTV Outdoor with Cap (Site A)*	45	520	34
*Photo-CCTV Outdoor with Sunglasses (Site A)*	41	490	31
***Photo-CCTV Totals***	**403**	**4030**	**334**
**Grand Totals**	**622**	**6220**	**334**

CCTV= closed-circuit television.

## Database utility and applications

A database of this scale can be utilised in a variety of training and research applications, particularly when considering this as the first database of facial images with such a large complement of facial photographs of African individuals. Among a variety of possible applications, the primary intended use is testing various methodologies and conditions for forensic facial comparison. Furthermore, this database can be utilised as a prime tool for training facial comparison experts to develop a high rate of competency and proficiency. Having an appropriate level of training is a crucial aspect of the judicial process ^1, 4^. In addition, the faces in this database could be used to generate stimuli for studies in psychology and marketing sciences relating to facial recognition. Likewise, the images in this database could be modified as required on an *ad hoc* basis and implemented to train and develop future machine learning and artificial intelligence systems for the purpose of facial recognition in a forensic context. Although a potentially laborious process, standardised landmarks could be added to allow for an estimated dimension calculation of the various facial proportions and features as required by facial recognition systems. However, the database is only available for *bona fide* researchers affiliated with academic research institutions and not for commercial use. An example use the dataset was designed for was validating the use of morphological analysis in forensic facial comparison across a photographic and CCTV sample ^27^. This was achieved by sub-setting selected photographs and CCTV recording stills from the database into facial image pools that were independently analysed ^27^ following the Facial Identification Scientific Working Group morphological analysis feature list ^42^.

## Challenges and limitations

Developing a database of facial photographs and surveillance footage is a logistically complex and tedious process. It can be difficult to recruit volunteers even in highly trafficked areas in a major university (the University of the Witwatersrand has over 39,500 students across five campuses), although people on a campus tend to feel more secure and therefore more willing to participate in this type of study. The recruitment and photography process require a large amount of manpower with a minimum of three to four volunteers needed per day as assistants for an efficient image acquisition process. The location management and site selection are also a highly demanding task as one needs to limit variations between recorded images as much as possible. This entire process needs to be carried out while still collecting images that are representative of a somewhat realistic scenario. The variations in conditions of recordings vary based on camera type, quality, and installation. The majority of CCTV recordings, for example, were collected in an outdoors area with sunny daylight conditions which is by design an actualistic sample, although inconsistent due to weather and lighting conditions changing throughout a given day.

Similarly, the photography collection was affected by uncontrollable varying lighting conditions for both the indoor and outdoor settings. These include dim lighting indoors leading to lower quality, higher noise images, and dappled light and sun position outdoors leading to highlights, shadow, and contrast artefacts. The objective to face distances were controlled as much as possible, without compromising the actualistic nature of the data, in order to minimise perspective distortion due to the nature of relaying a three-dimensional scene/object into a two-dimensional medium of photography ^43^. Despite the intent, at greater distances the focal plane of a camera lens requires adjusting and can result in lower clarity images ^43^, as is evident in the varied focal lengths of the CCTV cameras.

Furthermore, the nature of CCTV cameras depends on the entirety of the surveillance system installation. A variety of complications, including inconsistent IP camera network connectivity and power outages caused occasional complete data loss or footage corruption in the recording process, resulting in the overall reduced numbers of corresponding recordings. This data loss was particularly evident in the IP cameras, as in the particular set-up used by the university, they do not locally store footage but transfer it immediately to a central server. In the process, any interruption or fluctuation in local area network speeds or connectivity would result in data loss or corruption. Even though analogue cameras by default record at lower resolutions, the immediate local storage on a DVR device resulted in reduced data loss and no data corruption. In fact, a total of 710 participants were originally recruited and photographed and/or recorded. Following data loss, participants that requested to be excluded from the database and data corrupted during transfer from the CCTV cameras to the servers, only 622 of the 710 could be included in the database.

Male individuals were selected specifically as males are more commonly involved in criminal activity, both as victims and perpetrators, in both developed and developing nations ^44, 45^. However, other demographic factors such as age and ancestry were not strictly controlled, and sample composition resulted varied across all cohorts. This lack of strict control was primarily due to the sensitivity of labelling groups of individuals as belonging to specific descent groups, making requesting this information from participants one of the ethical limitations considered originally.

The intention of the authors is for this database to be further expanded to include female individuals, additional disguises in the form of make-up and face masks, as well as more variations of CCTV camera recordings, such as infrared night vision CCTV, to provide a better and more varied selection for its applications.

## Ethics of face databases

Overall, face databases are quite common with 27 photographic image databases available for use in the fields of head and face detection, tracking and recognition. These databases are usually created with specific criteria, such as particular facial expressions and lighting condition variations for testing a specific aspect of facial recognition. Amongst the 27 databases outlined in Table 1, seven have been collected from various media of personal photographs available on the internet ^7, 9, 11, 12, 15, 24, 37^, which is a legal yet perhaps ethically questionable practice, as the individuals included in these face databases have not provided consent for that inclusion. This is particularly common in the most recently developed databases (e.g. 7, 8, 15), most likely due to the broad availability of facial images and the highly efficient and accurate search engines. This practice can result in privacy-intrusive practices of freely available and commercialised application of facial recognition ^28^, which is an ever-increasing concern for the public with specific regard to privacy of data and images ^46^. The database compiled here is an example of a complex yet more ethical approach that limits commercialised application and potential research.

## Data availability

### Underlying data

The WFD is stored on the Wits Institutional Repository environment on DSpace ( WIReDSpace) and published under the following unique identifier: http://doi.org/10.17605/OSF.IO/WMA4C (this registration also contains the PhD study protocol that led to the development of the WFD and an addendum to the protocol registration highlighting the major changes to the methodological approach of the original protocol). A sample of the dataset is freely accessible at https://hdl.handle.net/10539/29924.

The database is an open access resource for use in strictly non-commercial research. In order to access the WFD, prospective users will have to apply for access to the Institutional Review Board overseeing ethical and scientific use of the database in order to safeguard the privacy and decency of the database’s participants. Once approved a researcher may use the database free of charge. Database access is restricted and limited to following the above-mentioned procedure, due to the nature of the data including potentially identifying information (facial physiognomy) of participants. In addition, strict limitations were imposed by the Human Research Ethics Committee (Medical) as well as the consent permissions agreed upon with the participants, which assign responsibility to the School of Anatomical Sciences to review access applications in ethical and scientific merit in order to exclusively conduct research. The access procedures and limitations are governed by a legally binding Conditions of Use document available on https://hdl.handle.net/10539/29924 ^40^ in conjunction to the freely accessible sample. Data will be made available to successful applicants under a temporary restricted licence guided by the aforementioned conditions of use document.

### Extended data

Open Science Framework: Wits Face Database: Description. https://doi.org/10.17605/OSF.IO/Q8V2R ^40^

This project contains the following extended data:

- Participant information sheet
- Participant consent form
- Conditions of use

Data are available under the terms of the Creative Commons Zero "No rights reserved" data waiver (CC0 1.0 Public domain dedication).
