# Supplementary material for: Development of the Wits Face Database: an African database of high-resolution facial photographs and multimodal closed-circuit television (CCTV) recordings
Source: F1000Res. 2021 Feb 19;10:131. [Version 1] doi: 10.12688/f1000research.50887.1 (PMC7986986; doi:10.12688/f1000research.50887.1)
Supplement: Peer review history

# Peer review: Reviewer response for version 1

**DOI:** 10.5256/f1000research.53979.r80712

**Published:** 2021-03-22

**Authors:** Won-Joon Lee

**Affiliations:** 1 Department of Forensic Medicine, National Forensic Service Seoul Institute, Seoul, South Korea

This manuscript stated the procedure and results of developing a facial image database from South African males. Also, the authors discussed the applicability and limitations of their research.

 This study is regarded to produce the best results from given conditions which they stated objectively in the section of challenges and limitations. It is considered that the database will benefit the research and researchers into facial recognition using CCTV images, especially South African males which available database has not been enough in this field.  

 Consequently, I would like to recommend this manuscript to be indexed.

Are sufficient details of methods and materials provided to allow replication by others?

Yes

Is the rationale for creating the dataset(s) clearly described?

Yes

Are the datasets clearly presented in a useable and accessible format?

Yes

Are the protocols appropriate and is the work technically sound?

Yes

Reviewer Expertise:

Craniofacial Identification

I confirm that I have read this submission and believe that I have an appropriate level of expertise to confirm that it is of an acceptable scientific standard.

# Peer review: Reviewer response for version 1

**DOI:** 10.5256/f1000research.53979.r79950

**Published:** 2021-03-05

**Authors:** Ching-Yiu Jessica Liu

**Affiliations:** 1 Liverpool John Moores University, Liverpool, UK

This article described the development of the 'Wits Face Database'. The authors collected CCTV footage from three different environments, along with standardised and uncontrolled facial photographs detailed in Table 2. A total of 622 adult male individuals with facial anthropological features resembling South African descent were recruited.

 It will be beneficial to document the framerates of the CCTV recordings. 

 The mean height for black South African males was used for the photography station for ST images. Did you have to make any adjustments for different heights or were all participants within the capture range?

 "individuals with facial anthropological features resembling South African descent were recruited." I am not sure I fully understand what this includes and excludes. You mentioned that age and ancestry were not strictly controlled along with the sensitivity of labelling groups. Does this mean anyone who was an adult male was recruited?

Are sufficient details of methods and materials provided to allow replication by others?

Yes

Is the rationale for creating the dataset(s) clearly described?

Yes

Are the datasets clearly presented in a useable and accessible format?

Yes

Are the protocols appropriate and is the work technically sound?

Yes

Reviewer Expertise:

Facial Anthropology

I confirm that I have read this submission and believe that I have an appropriate level of expertise to confirm that it is of an acceptable scientific standard.

# Response

**Published:** 2021-03-17

**Authors:** Nicholas Bacci

**Affiliations:** University of the Witwatersrand, South Africa

Dear Dr Liu,

 Thank you for your prompt and positive review. We appreciate your insights for the missing information and points requiring clarification. We will be including the following information in a new version of the manuscript to provide the information to all readers:

1. Comment: It will be beneficial to document the framerates of the CCTV recordings. Response: The framerates and codec information for the IP camera CCTV recordings will be included. However, the analogue camera did not store framerate or codec information in its metadata or the manufacturer website. Amendment: The following statements will be included in the revised manuscript version for clarity: “The recordings from these two cameras were encoded in MPEG-H Part2/HEVC (H.265) codec and had a frame rate of 20 frames per second.”. “No codec and framerate information was available for this camera both on the manufacturer website and the recording metadata.”
2. Comment: The mean height for black South African males was used for the photography station for ST images. Did you have to make any adjustments for different heights or were all participants within the capture range? Response: The height of the tripod for the ST and WT photographs was not adjusted, it was maintained fixed at the average height of black South African males throughout the data collection process. Only a handful of participants were significantly above or below the mean height in order to affect the composition of the photographs. In those cases the angle of the camera was adjusted slightly in the vertical axis to retain the same composition. Amendment: The following clarification will be included in the manuscript: “In all cases, for both ST and WT photographs, the tripod height was not adjusted and left at the fixed height of 1600mm. The majority of participants were within the capture range; however, for a few of the individuals who were either vastly below or above average South African male height, the camera was tilted in the vertical axis of the tripod in order to align their eyes along the uppermost 1/3rd of the portrait composition. No other changes were made in terms of focal length or post crop editing.”
3. Comment: "individuals with facial anthropological features resembling South African descent were recruited." I am not sure I fully understand what this includes and excludes. You mentioned that age and ancestry were not strictly controlled along with the sensitivity of labelling groups. Does this mean anyone who was an adult male was recruited? Response: Clarification on the participants included in the study will be included. Male South Africans of facial appearance resembling African descent were specifically recruited for the database. Amendment: The statement speaking to participant descent will be amended for clarity as follows: “Potential participants were identified and recruited near the data collection sites based on facial anthropological features resembling South African males of African descent and being older than 18 years of age.”.

Best Regards,

 Nicholas Bacci
